# Instruments—Pluggers

**Published:** 1870-04

**Authors:** 


### INSTRUMENTS—PLUGGERS.

We have in use a set of Varney's Pluggers, manufactured by S. S. White, and it is difficult for us to decide which possesses the greatest merit, the excellence of the forms, or the perfection of the manufacture. The forms are particularly adapted to filling the front teeth, though they may be used for filling molars and bicuspids—they are well adapted for filling all proximal cavities. The neatness, delicacy, perfection of finish, and temper are not excelled by any similar instruments in the market; and for the purposes specified, there are no forms in the market equal to them. There are two instruments in this set we would not be deprived of for the price of the whole, $20. The progress made in the manufacture of these instruments is truly astonishing. Though we can hardly see where there is room for it, yet we doubt not there will be higher points of excellence attained; and it will be our joy to herald abroad to the widest extent, every step of progress made. And may the finely wrought minds, of sharp perception, and fruitful invention devise and plan; and keen eyes and skillful hands execute, till perfection shall be the reward.

